# Lens Injury Has a Protective Effect on Photoreceptors in the RCS Rat

**DOI:** 10.1155/2013/814814

**Published:** 2013-09-19

**Authors:** Peter Heiduschka, Daniel Renninger, Dietmar Fischer, Adrienne Müller, Sabine Hofmeister, Ulrich Schraermeyer

**Affiliations:** ^1^Department of Ophthalmology, University of Münster Medical School, Domagkstraße 15, 48149 Münster, Germany; ^2^Centre for Ophthalmology, Experimental Vitreoretinal Surgery, Schleichstraße 12/1, 72076 Tübingen, Germany; ^3^Experimental Neurology, Department of Neurology, Heinrich Heine University, Life Science Centre, Merowingerplatz 1a, D-40225 Düsseldorf, Germany; ^4^Experimental Neurology, University Ulm, Albert-Einstein-Allee 11, 89081 Ulm, Germany

## Abstract

Lens injury induced activation of retinal glia, and subsequent release of ciliary neurotrophic factor (CNTF) and leukaemia inhibitory factor (LIF) potently protect axotomised retinal ganglion cells from apoptosis and promotes axon regeneration in the injured optic nerve. The goal of the current study was to investigate if similar effects may also be applicable to rescue photoreceptors from degeneration in a model of retinitis pigmentosa. Lens injury was performed in the Royal College of Surgeons (RCS) rats at the age of one month. The survival of photoreceptors was evaluated histologically, and retinal function was analysed by electroretinography (ERG). Expression of CNTF was also analysed. Lens injury significantly enhanced the survival of photoreceptors 1 month after surgery compared to untreated controls, which was associated with an enhanced ERG response. In addition, lens injury significantly protected photoreceptors from degeneration in the contralateral eye, although to a much lesser extent. We could show that lens injury is sufficient to transiently delay the degeneration of photoreceptors in the RCS rat. The observed neuroprotective effects may be at least partially mediated by an upregulation of CNTF expression seen after lens injury.

## 1. Introduction

It has recently been shown that lens injury or intravitreal applications of lens-derived *β*/*γ*-crystallins potently protect axotomised retinal ganglion cells (RGCs) from cell death and stimulate axon regeneration in the injured optic nerve [1–5]. All treatments induce an activation of retinal astrocytes and Müller cells, which subsequently upregulate and release the cytokines ciliary neurotrophic factor (CNTF) and leukaemia inhibitory factor (LIF) [6–10]. The strong neuroprotective effects of lens injury are completely absent in CNTF/LIF double knockout mice, demonstrating that both cytokines act as essential key mediators [9, 11]. Similar to these factors, interleukin 6 also promotes neuroprotection [12]. 

The purpose of the current study was to investigate whether lens injury is also sufficient to protect photoreceptors from degeneration. As a model, we chose the Royal College of Surgeons (RCS) rat, which is a commonly used model of retinal degeneration, in particular retinitis pigmentosa. The cells of the retinal pigment epithelium (RPE) are not capable of phagocytosing photoreceptor outer segments due to a mutation in the Mertk gene. This leads to a gradual degeneration of the photoreceptors starting at the time point of eye opening (i.e., approximately at P20) and being completed at the age of 3 months [13, 14]. Mutations in the Mertk gene have also been discovered in retinitis pigmentosa patients [15, 16]. Our results demonstrate that lens injury induces an upregulation of CNTF in retinal astrocytes and Müller cells. Moreover, it reduced the cell death of photoreceptors and was correlated with a rescue of retinal function, which was evaluated by electroretinography (ERG).

## 2. Materials and Methods

### 2.1. Animals

The RCS rats were bred in our animal facility. They received food and water *ad libitum* and were kept at dimmed light in a 12 hours/12 hours light/dark cycle. All experimental procedures conducted on the animals were carried out in accordance with the ARVO statement for Use of Animals in Ophthalmic and Vision Research.

### 2.2. Surgery

Lens injury was performed using a microscope with illumination in 14 RCS rats at the age of one month, shortly after the onset of retinal degeneration. Eight animals were used for histology, two for immunohistochemistry, and four for Western blot analysis. Animals were anaesthetised by a mixture of ketamine and xylazine (120 mg/kg ketamine, 10 mg/kg xylazine). A small incision was made into the temporal corner of the eyes. The eyeball was rotated into the nasal direction, and the conjunctiva was incised to allow direct access to the sclera. A sharp 25-gauge injection needle was used to puncture the lens by inserting it into the eye through the sclera. In order to assure injury of the lens, the needle was rotated two or three times inside the lens before retracting it. The success of lens penetration was controlled by a funduscopic inspection of the eye. After lens injury, the eyeball was brought back into its normal position, and the eye was covered by antibiotic ointment (Gentamytrex, Dr. Mann Pharma, Berlin). Finally, the animals were kept at 37°C on a heating pad until waking up and then brought back into their cages.

### 2.3. Histology

Four RCS rats were killed 1 month after the lens injury and four RCS rats after two months. In addition, four untouched animals were used as controls for the ages of two and three months. Consequently, four eyes were available for histological analysis for each age and each experimental group (control, lens injury, and contralateral to lens injury).

**Figure 1:**
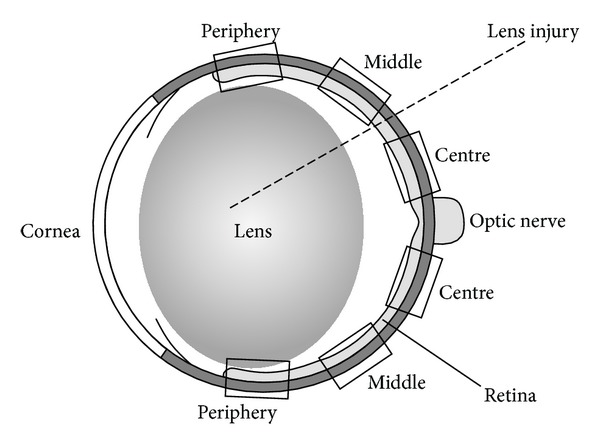
Scheme of the rat eye. The regions where evaluation of photoreceptor survival was performed are indicated.

The animals were enucleated, the eyes were fixed in formalin, embedded in paraffin wax, and haematoxylin-stained sections were prepared according to standard procedures. Digital images were taken, and photoreceptor nuclei per 100 *μ*m length of retinal section were counted manually. Evaluation was performed in central, middle, and peripheral regions of the retina, as indicated in Figure 1. Five sections obtained in the region of the optic nerve head were evaluated per eye. The investigator who performed the evaluation of photoreceptor survival was blinded to the arrangement and identity of the sections to prevent the introduction of bias to the analysis.

### 2.4. Immunohistochemistry

In order to check expression of CNTF after lens injury in RCS rat eyes, paraffin sections of the eyes were deparaffinised using standard procedures. The sections were heated for 3 minutes in Tris buffer/EDTA (pH 9) and blocked with BSA (1% in PBS) for 1 hour. A double staining was performed applying a mixture of primary antibodies against CNTF (goat polyclonal anti-CNTF antibody, Santa Cruz Biotechnology, Santa Cruz, CA, USA, dilution 1 : 100) and the glial fibrillary acidic protein (GFAP, rabbit polyclonal anti-GFAP antibody; DakoCytomation, Glostrup, Denmark, dilution 1 : 4000) at 37°C for 2 hours. The sections were washed three times with TBS and Tween 20. A mixture of secondary antibodies (Alexa Fluor 488-labelled donkey anti-rabbit IgG antibody for GFAP, Invitrogen Corporation, Carlsbad, California, dilution 1 : 1000, and Cy3-labelled AffiniPure donkey anti-goat IgG antibody for CNTF, Jackson ImmunoResearch Laboratories, West Grove, Pennsylvania, dilution 1 : 400) was applied for 45 minutes at room temperature. After washing with distilled water, the samples were embedded in FluorSave (Calbiochem, Darmstadt, Germany), coated with a cover glass slide, and inspected using a fluorescent microscope.

### 2.5. Western Blot Analysis

Retinas were isolated 5 days or 1 month after a lens injury and frozen in liquid nitrogen before further processing. In addition, eyes from untreated animals and the eyes contralateral to the lens injury were analysed. Retinal tissue of two eyes was pooled in each group. Isolated retinas were transferred into lysis buffer (20 mM Tris/HCl pH 7.5, 10 mM KCl, 250 mM sucrose, 10 mM NaF, 1 mM DTT, 0.1 mM Na_3_VO_4_, 1% TritonX-100, and 0.1% SDS) with 1/100 protease inhibitor (Calbiochem, CA, USA). Retinas were homogenised and centrifuged at 5,000 rpm for 10 min at 4°C. The supernatants were analysed by Western blot assay. Separation of proteins was performed by 10% sodium-dodecyl-sulfate-polyacrylamide gel electrophoresis (SDS-PAGE), according to standard protocols (Bio-Rad, Hercules, USA). After SDS-PAGE, proteins were transferred to nitrocellulose membranes (Amersham, UK). The blots were blocked either in 5% dried milk or in 2% ECL Advance blocking agent in Tris-buffered saline-Tween 20 (TBS-T). They were then processed for immunostaining with either a polyclonal antibody against rat CNTF (Serotec, 1 : 5000) or an antibody against tubulin (Babco, Richmond, CA, 1 : 2000) at 4°C overnight. Bound antibodies were visualised with anti-rabbit or anti-mouse immunoglobulin G (IgG) secondary antibodies conjugated with horseradish peroxidase diluted at 1 : 80,000 (all Sigma, St. Louis, USA). The antigen-antibody complexes were detected by enhanced chemiluminescence (ECL, Amersham, Buckinghamshire, UK).

### 2.6. Electroretinography

ERG analysis was performed in all RCS rats listed in the histology paragraph at the ages of two and three months. Animals were dark adapted over a period of at least 24 hours. They were anaesthetised by an intraperitoneal injection of a mixture of ketamine and xylazine (120 mg/kg ketamine, 10 mg/kg xylazine). The corneas of the eyes of the anaesthetised animals were desensitised with Novesine (Novartis Ophthalmics). The animals were placed on a heated platform (37°C) to keep their body temperature constant during the measurements. Gold wire ring electrodes placed on the corneas of both eyes served as working electrodes. A gold wire ring electrode was placed in the mouth to serve as a reference electrode. The pupils were dilated with Tropicamide (Novartis Ophthalmics). Standard electroretinographic measurements were performed using the commercial RetiPort32 device from Roland Consult Systems (Brandenburg, Germany), with scotopic flash ERG at light intensities of 0.0003 and 100 cd·s/m², an additional run for scotopic oscillatory potentials, photopic flash ERG after 10 minutes of light adaptation, and photopic oscillatory potentials. The light intensity used for the flashes in the photopic ERG measurements was 100 cd·s/m². The analogue filters of the ERG device were set to the frequency ranges of 0.5 to 200 Hz for both scotopic and photopic flash ERG and 50 to 500 Hz for oscillatory potentials.

ERG measurements were performed simultaneously on both eyes to compare lens injury treated and contralateral eyes, and the performing person did not know which eyes had been lens-injured.

## 3. Results

**Figure 2:**
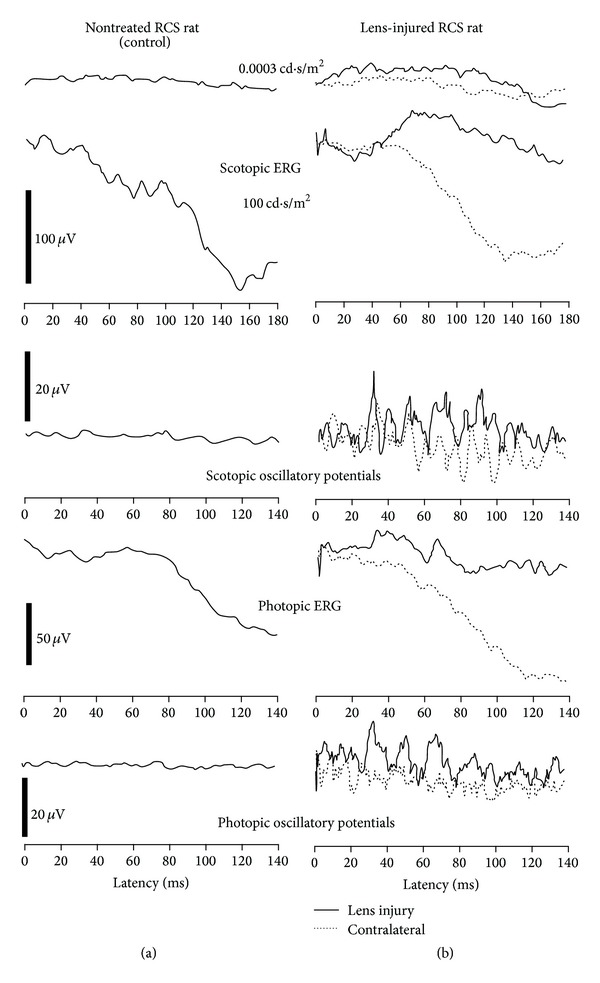
Typical electroretinographic waveforms recorded in 2-month-old RCS rats. The waveforms obtained in the control rat without treatment are shown in the left column, and the waveforms of the lens-injured animal are shown in the right column. The solid lines represent the waveforms recorded in the lens-injured eye, whereas dotted lines represent waveforms recorded in the contralateral eye. For the scotopic flash ERGs, only two traces are shown to be obtained at light intensities as indicated, one for a pure rod ERG and one for maximal mixed rod-cone response. Please note the different scales.

The ERG waveforms obtained at the age of 2 months, that is, one month after lens injury, are shown in Figure 2. In the left column, the ERG waveforms of a nontreated RCS rat are shown. The ERG waveforms obtained in an RCS rat after lens injury are shown in the right column. The shapes of the curves obtained in the contralateral eye show that degeneration in 2-month-old rats has proceeded to an extent that virtually no retinal activity could be recorded (dotted lines). By contrast, some residual activity was detected in the lens-injured eye (solid lines), which was the case in three out of four rats. It was particularly visible in the scotopic and photopic ERGs and to a lesser extent in the scotopic and photopic oscillatory potentials. In the three rats where a response could be obtained, amplitudes at maximum light intensity (100 cd·s/m^2^) were as follows: scotopic a-wave 18.3 ± 8.3 *μ*V, scotopic b-wave 30.5 ± 13.0 *μ*V, scotopic oscillatory index (sum of the amplitudes of the first four oscillations) 67.6 ± 28.9 *μ*V, photopic b-wave 13.7 ± 3.8 *μ*V, and photopic oscillatory index 33.1 ± 3.8 *μ*V.

No ERG response was found in lens-injured eyes 2 months after lens injury (not shown).

**Figure 3:**
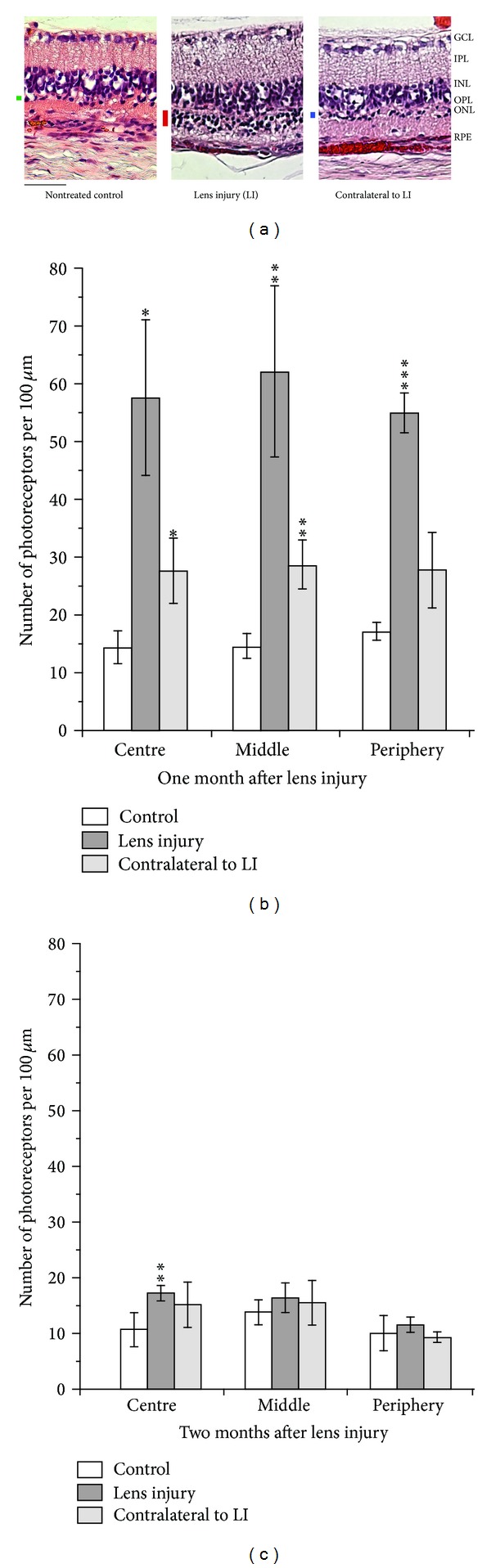
(a) Histological sections of retinas of 2-month-old RCS rat eyes. From left to right, the retina of an untouched animal (nontreated control), a retina where the lens was injured, and a retina of an eye contralateral to lens injury are shown. The black bars indicate the thickness of the outer nuclear layer. Scale bar: 50 *μ*m. (b), (c) Numerical evaluation of the numbers of photoreceptor nuclei in the eyes of RCS rats: untouched animals (nontreated control), eyes with lens injury, and contralateral eyes. Age of the animals: (b) 2 months and (c) 3 months. Mean values are shown, with the error bars indicating standard deviations. Significance of difference compared to vehicle injection: *P < 0.05, **P < 0.01, and ***P < 0.001.

After the ERG measurements, the animals were enucleated, and paraffin sections of the eyes were prepared. We compared paraffin sections of RCS rat eyes after lens injury, corresponding contralateral eyes, and eyes of untreated age-matched control animals. Representative histological sections are shown in Figure 3(a). The bars indicate localisation and thickness of the outer nuclear layers.

Compared to untreated control groups, significantly more photoreceptors survived in the lens-injured eyes in all three regions of the retina that were separately evaluated (Figure 3(b)). Surprisingly, untreated eyes contralateral to the ones exposed to lens injury also revealed a slight but significant increase in the number of photoreceptors compared to untreated controls (Figure 3(b)). In 3-month-old RCS rats where the degeneration of photoreceptors has progressed further, a slight but still significantly higher survival of photoreceptors was detected only in the central region of lens-injured eyes and not elsewhere (Figure 3(c)).

**Figure 4:**
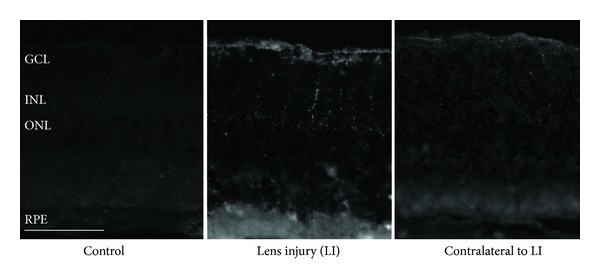
Immunohistochemical staining against CNTF of retinal paraffin sections of a 2-month-old lens-injured RCS rat. In the lens-injured eye, CNTF-positive cells are visible mainly in the ganglion layer. Some photoreceptor nuclei with CNTF immune reactivity were also found. Scale bar: 50 *μ*m. GCL: ganglion cell layer, IPL: inner plexiform layer, INL: inner nuclear layer, ONL: outer nuclear layer, and PR OS: photoreceptor outer segments.

We analysed the eyes immunohistochemically for CNTF expression (Figure 4). In contrast to nontreated control animals, which showed virtually no CNTF immunoreactivity, a clearly upregulated CNTF immunoreactivity was found in the retinas of lens-injured eyes in the nerve fibre and ganglion cell layers, and also occasionally in deeper layers of the retina, including the outer nuclear layer. The CNTF immunoreactivity was considerably weaker in the contralateral eyes, though showing a similar pattern.

**Figure 5:**
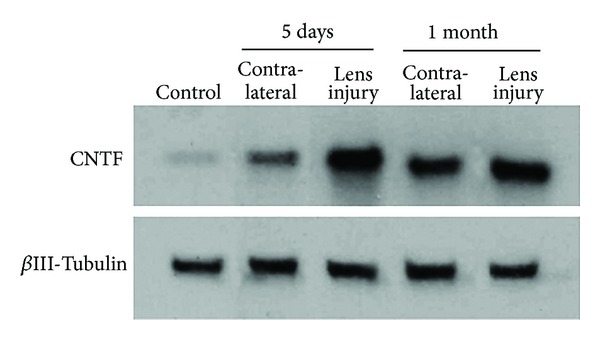
Result of the Western blot analysis for CNTF in retinal samples obtained from lens-injured eyes and contralateral eyes five days and one month after lens injury. As a measure for the applied sample size, *β*III-tubulin was analysed simultaneously.

Finally, the lens-injury induced upregulation of retinal CNTF expression was confirmed by Western blot analysis for CNTF, showing enhanced CNTF levels in lens-injured eyes, and, to a lesser extent, also in the contralateral eyes (Figure 5).

## 4. Discussion

Since the discovery of the significant neuroprotective and neuroregenerative effects of lens injury on damaged retinal ganglion cells, this experimental paradigm has been successfully applied by several groups [1–4, 6, 9, 10, 17–21]. We here extended the application of lens injury to the protection of dying photoreceptors, using the RCS rat as a well-established and widely used model of inherited retinal degeneration. In this strain, photoreceptors start to degenerate shortly after the new-born animals open their eyes, and no photoreceptors are left at the age of 3 months [13].

As shown, a significantly higher portion of photoreceptors survived in lens-injured eyes in 2-month-old rats, whereas no neuroprotection was found in 3-month-old rats, suggesting that the lens injury effects are only effective for a limited time period. This is consistent with the idea that crystallins are released from the injured lens and subsequently stimulate retinal glial cells. After stimulation, the endogenous CNTF levels return to basal levels and, therefore, can no longer protect the photoreceptors [6, 9, 18, 22]. 

Consistently, we found CNTF immunoreactivity mainly in the nerve fibre and ganglion cell layers and additionally in deeper layers of the retina. Comparison with the GFAP immunoreactivity suggests that CNTF is produced by astrocytes and Müller cells (not shown). Moreover, we have also seen occasional CNTF immunoreactivity in the outer nuclear layer indicating that even a few photoreceptors themselves could probably produce or bind CNTF upon stimulation by lens injury. A major role of CNTF in mediating the neuroprotective effects of lens injury on photoreceptors is also supported by previous reports, demonstrating that CNTF is neuroprotective to photoreceptors in different animal models of retinal degeneration [23, 24]. 

Another interesting finding of the current study was that moderate neuroprotective effects were also found in the eyes contralateral to the lens-injured eye. This observation was in line with a noticeable CNTF immunoreactivity in the contralateral eyes. Mutual influences between the two eyes have been known for a number of years [25]. The mechanisms, however, are not clear so far, and further research is required to understand this phenomenon in detail. 

We performed electroretinographic measurements in the RCS rats to check if lens injury resulted in an improved retinal function. In the eyes contralateral to lens injury, no retinal response to light stimulation could be measured. The scotopic and photopic ERG waveforms showed a negative slope, which is typical for RCS rats with progressed photoreceptor degeneration [26]. Consequently, although we have seen slight morphological protection in the contralateral eyes in 2-month-old rats, it was not sufficient to record even a residual retinal function.

By contrast, an ERG response could be measured in three out of four lens-injured eyes in 2-month-old rats. Obviously, the extent of protection in these eyes was sufficient to maintain a residual retinal activity. Whether a function of photoreceptors is preserved or not seems to depend on the amount of surviving photoreceptors. The one lens-injured eye where we could not detect any improvement of the ERG response was also the eye with the smallest morphological effect, that is, with the smallest number of surviving photoreceptors. We speculate that there could be a kind of “bystander” effect between the photoreceptors, enabling them to respond considerably to light as long as there is a sufficient number of them. 

Lens injury causes cataract and, consequently, opacity of the lens that hinders access of light to the retina. It is thus all the more noteworthy that we could record an enhanced ERG response at all one month after lens injury in three out of four lens-injured animals compared to the contralateral eyes with their nonopaque lenses. 

Two months after lens injury, the protective effects were almost completely lost. Corresponding to the poor, that is, no longer existing morphological effects of lens injury, we could not detect any differences in the ERG response between lens-injured and contralateral eyes. 

In summary, we conclude that lens injury exerts protective effects on photoreceptors in the RCS rat as a model of retinal degeneration. As this treatment does not address the actual defect in the RCS rat, the protective effect is only moderate and transient. Nevertheless, we propose that lens injury, or injection of lens crystallins as an alternative, could be a useful option for an accompanying treatment during gene therapy or other therapeutical approaches.

## References

[1] Fischer D, Pavlidis M, Thanos S (2000). Cataractogenic lens injury prevents traumatic ganglion cell death and promotes axonal regeneration both in vivo and in culture. *Investigative Ophthalmology and Visual Science*.

[2] Leon S, Yin Y, Nguyen J, Irwin N, Benowitz LI (2000). Lens injury stimulates axon regeneration in the mature rat optic nerve. *The Journal of Neuroscience*.

[3] Fischer D, Heiduschka P, Thanos S (2001). Lens-injury-stimulated axonal regeneration throughout the optic pathway of adult rats. *Experimental Neurology*.

[4] Lorber B, Berry M, Logan A (2005). Lens injury stimulates adult mouse retinal ganglion cell axon regeneration via both macrophage- and lens-derived factors. *European Journal of Neuroscience*.

[5] Fischer D, Hauk TG, Müller A, Thanos S (2008). Crystallins of the *β*/*γ*-superfamily mimic the effects of lens injury and promote axon regeneration. *Molecular and Cellular Neuroscience*.

[6] Müller A, Hauk TG, Fischer D (2007). Astrocyte-derived CNTF switches mature RGCs to a regenerative state following inflammatory stimulation. *Brain*.

[7] Joly S, Lange C, Thiersch M, Samardzija M, Grimm C (2008). Leukemia inhibitory factor extends the lifespan of injured photoreceptors in vivo. *The Journal of Neuroscience*.

[8] Bürgi S, Samardzija M, Grimm C (2009). Endogenous leukemia inhibitory factor protects photoreceptor cells against light-induced degeneration. *Molecular Vision*.

[9] Leibinger M, Müller A, Andreadaki A, Hauk TG, Kirsch M, Fischer D (2009). Neuroprotective and axon growth-promoting effects following inflammatory stimulation on mature retinal ganglion cells in mice depend on ciliary neurotrophic factor and leukemia inhibitory factor. *The Journal of Neuroscience*.

[10] Müller A, Hauk TG, Leibinger M, Marienfeld R, Fischer D (2009). Exogenous CNTF stimulates axon regeneration of retinal ganglion cells partially via endogenous CNTF. *Molecular and Cellular Neuroscience*.

[11] Fischer D (2010). What are the principal mediators of optic nerve regeneration after inflammatory stimulation in the eye?. *Proceedings of the National Academy of Sciences of the United States of America*.

[12] Leibinger M, Müller A, Gobrecht P, Diekmann H, Andreadaki A, Fischer D (2013). Interleukin-6 contributes to CNS axon regeneration upon inflammatory stimulation. *Cell Death and Disease*.

[13] Dowling JE, Sidman RL (1962). Inherited retinal dystrophy in the rat. *The Journal of Cell Biology*.

[14] D’Cruz PM, Yasumura D, Weir J (2000). Mutation of the receptor tyrosine kinase gene Mertk in the retinal dystrophic RCS rat. *Human Molecular Genetics*.

[15] Gal A, Li Y, Thompson DA (2000). Mutations in MERTK, the human orthologue of the RCS rat retinal dystrophy gene, cause retinitis pigmentosa. *Nature Genetics*.

[16] Brea-Fernández AJ, Pomares E, Brión MJ (2008). Novel splice donor site mutation in MERTK gene associated with retinitis pigmentosa. *British Journal of Ophthalmology*.

[17] Lorber B, Berry M, Logan A, Tonge D (2002). Effect of lens lesion on neurite outgrowth of retinal ganglion cells in vitro. *Molecular and Cellular Neuroscience*.

[18] Lorber B, Berry M, Logan A (2008). Different factors promote axonal regeneration of adult rat retinal ganglion cells after lens injury and intravitreal peripheral nerve grafting. *The Journal of Neuroscience Research*.

[19] Yin Y, Cui Q, Li Y (2003). Macrophage-derived factors stimulate optic nerve regeneration. *The Journal of Neuroscience*.

[20] Berry M, Ahmed Z, Lorber B, Douglas M, Logan A (2008). Regeneration of axons in the visual system. *Restorative Neurology and Neuroscience*.

[21] Hauk TG, Müller A, Lee J, Schwendener R, Fischer D (2008). Neuroprotective and axon growth promoting effects of intraocular inflammation do not depend on oncomodulin or the presence of large numbers of activated macrophages. *Experimental Neurology*.

[22] Lorber B, Berry M, Douglas MR, Nakazawa T, Logan A (2009). Activated retinal glia promote neurite outgrowth of retinal ganglion cells via apolipoprotein E. *The Journal of Neuroscience Research*.

[23] Kassen SC, Thummel R, Campochiaro LA, Harding MJ, Bennett NA, Hyde DR (2009). CNTF induces photoreceptor neuroprotection and Müller glial cell proliferation through two different signaling pathways in the adult zebrafish retina. *Experimental Eye Research*.

[24] Kent TL, Glybina IV, Abrams GW, Iezzi R (2008). Chronic intravitreous infusion of ciliary neurotrophic factor modulates electrical retinal stimulation thresholds in the RCS rat. *Investigative Ophthalmology and Visual Science*.

[25] Bodeutsch N, Siebert H, Dermon C, Thanos S (1999). Unilateral injury to the adult rat optic nerve causes multiple cellular responses in the contralateral site. *Journal of Neurobiology*.

[26] Bush RA, Hawks KW, Sieving PA (1995). Preservation of inner retinal responses in the aged royal college of surgeons rat: evidence against glutamate excitotoxicity in photoreceptor degeneration. *Investigative Ophthalmology and Visual Science*.

